# Cataphoresis—Early History*International Dent. Jour., Oct,. 1896.

**Published:** 1897-01

**Authors:** J. M. Walker


### Monthly Digest.

Prepared for the Dental Register by Mrs. J. M. Walker.

### Cataphoresis—Early History.*

Dr. D. F. McGraw gives some extracts from a paper read before several Societies in 18?8 (to be found in *Dental Review*, February, 1889), showing his use, at that time, of the cataphoric

*International Dent. Jour., Oct,. 1896.

or electrolytic process in obtunding sensitive dentine, the solution used being equal parts of a 12-percent solution of cocaine and absolute alcohol, used with the galvanic-current. His deductions as to the physiological effects of the process were as follows:

"That the galvanic-current acts as a vehicle for conducting the medicinal agents; that the cocaine anæsthetizes the odontoblastic cells and the pulps; that the styptic properties of the alcohol act upon the dentinal fibrils, they being of an albuminous nature, causing contraction and increased density and firmness."

In the treatment of peridental inflammation he used a stronger current with a fluid containing seven ounces of saturated solution chloride of sodium to one ounce tincture of ergot. For the removal of pulps he recommended that inflammation and congestion be first reduced, in order to obtain full anæsthetic effects.

Dr. Iredell, in a paper on "Painless Dentistry," read before the New Jersey State Dental Society,* finds the forerunner of modern cataphoresis in what was known as "Voltaic Narcotism," on which a report was made in February, 1859, the committee reporting that, while the application of a narcotic solution to a tooth, in connection with a continuous current of electricity of considerable intensity, leads, unquestionably, to the production of local anæsthesia, it can be used successfully only in cases where the cavity of the tooth is exposed, the time required for its application being also a serious obstacle to its practical usefulness, the pain produced in the first stages being also always a source of complaint. The committee expressed the opinion that important improvements might be made in the process.

In November of the same year, Dr. Williams, of Buffalo, having made various improvements in the apparatus then employed, asserted that, if properly applied, electricity is a perfect local anæsthetic in almost every case, and more uniform in its action than any other agent heretofore used, either locally or otherwise, great care and delicacy, however, being necessary in introducing the current—upon that depends success.

To the facilties enjoyed at that time cocaine has been added,

* Items of Interest, October, 1896.

this medicament losing its most dangerous feature—its action upon the heart—when forced into the tissue by the aid of electricity.

Dr. Iredell reviews the various cocaine combinations—sulphuric acid, guaiacol, aconite and chloroform, etc., and concludes that a 20-percent solution of cocaine, made slightly saline, affords the most satisfactory results, electricity being thus readily conducted, the medicament also flowing quickly into the tissues.

In the report of a clinical demonstration before the British Dental Association,* H. B. Ezard, L. D. S., is quoted in reference to his experiments of eight years ago in the endeavor to electrically inject cocaine into sensitive dentine, abandoned at that time because he was unable to procure or manufacture a method of reducing the current to zero, an object since attained by others in the different current-controllers now in use.

He now uses the Wheeler Volt-Selector, using a 20-percent solution of cocaine hydrochloride in guaiacol, disguising the "abominable and penetrating odor" of the latter by adding one part ottar of roses, or one part heliotropin to two parts of the guaiacol-cocaine solution.

He does not advise the use of the guaiacol-cocaine for extraction, because of its too-strongly escharotic action on the mucous membrane. He uses it especially for the "immediate treatment" of exposed, putrescent and aching pulps, cataphorizing the pulp and covering it with a transparent celluloid cap, over which he fills "right off." He argues that if you mummify and embalm the pulp and then seal it up, you can not have any more trouble.

ELECTRICITY AND ITS APPLICATIONS IN DENTISTRY. †

Messrs. W. E. Clark and Philip W. Davis, in a paper read before the American Academy of Dental Science, reviewed, comprehensively, the subject of Electricity, beginning in its infancy with the bits of amber brought by the Phœnician traders from the shores of the Baltic, and worn by the Greeks as amulets because of its mysterious power of attraction—the scientific study of frictional electricity as far back as the sixteenth century; the

[illegible] Dental Cosmos, Oct., 1896.

[illegible]ternational Dental Journal, Nov., 1896.

discovery of the Leyden jar in 1748 ; the invention of the electric battery in the middle of the eighteenth century ; the modern dynamo and its construction.

The advent of the incandescent lamp placed the electric current on tap, so to speak, for any and every purpose, and the way to improve his method was put within the reach of the progressive dentist.

With sufficient knowledge and proper apparatus there are very few processes connected with dentistry which may not be benefited by the use of electricity. Light and heat, the dental engine, the laboratory motor, the electric furnace, soldering iron, electric fans, mouth lamps, hot-air syringe, hot-water heater, root-dryer, cautery, gold-annealer, and lastly, local anæsthesia by cataphoresis. With these acids better work can be done with greater convenience, in less time, commanding larger remuneration.

Dr. H. W. Gillett, in a paper read at the same meeting, defines certain technical terms necessary to be understood in the consideration of the application of electricity to dentistry.

The term, *coulomb*—the unit of quantity—corresponds to cubic feet of gas ; *ampere*, the current-strength, corresponds to cubic feet (per second) of gas flow, measured by thousandths of an ampere—milliamperes. The *volt*, the unit of force, corresponds to pounds-pressure (per square inch) in gas. The resistance is measured in *ohms*.

As stated by Messrs. Clark and Davis in the preceding paper, " the pressure in volts, divided by the resistance in ohms, equals the quantity in amperes."

Cataphoresis is the term most commonly used in discussing the process of carrying medicaments into the tissues of the human body by means of the electric-current, a process of recognized value to the electro-therapeutist. The current from any set of primary batteries (or any continuous-current lighting system, provided the current be steady), answers the requirements.

A dry-battery outfit has the merit of steadiness of current and of needing little attention in caring for it. For dental uses it is necessary to have an apparatus by which the current can be very thoroughly controlled, the living tooth being much more sensitive to the irritation of the electric-current than most other tissues of the human body. Given the ability to control the current (increasing by not more than ¼-volt steps, registered by a suitable volt-index), anæsthetics can be forced into dentine, and its sensitiveness decreased or entirely annihilated with very little, if any, discomfort to the patient. It is also desirable that the apparatus should have a milliampere-meter to register the amount or quantity of current used. The tooth to be operated upon should be insulated by the rubber-dam, and any metal-fillings also insulated by covering with soft gutta-percha, wax, varnish, etc. A pellet of cotton, loosely rolled and of suitable size and shape to about fill the cavity, should be saturated with a freshly-made 20 or 25-percent aqueous solution of cocaine and placed in the cavity. (Dr. Brackett* uses a much stronger solution of cocaine, placing a little mass of the pure crystals on a tile, and adding a little water, making, probably, a 30 or 40-percent solution.)

The sponge of the negative electrode should be well wet with water or a dilute salt-solution. It may be held in the hand by the patient or applied to the side of the face or neck by an attendant. With needle standing at zero, apply the positive electrode to the cotton in the cavity, and turn on the current, increasing it slowly and cautiously, watching the patient, and stopping at the least indication of pain, adding only as it can be borne without real discomfort. The strength of current that is borne, and the time necessary to obtain the anæsthetic effect, varies greatly with different patients. After the cocaine effect is reached the current can be increased more rapidly up to thirty or forty volts, if necessary fifteen or twenty being an average application, which may be obtained in from seven to fifteen minutes, though forty-five or fifty minutes may be required in extreme cases. When the application is complete, turn the current back nearly to zero and remove the negative electrode first. If the patient is not very sensitive to the current, it may not be necessary to turn back to zero, but merely slide the negative off slowly, breaking contact gradually. It may be necessary

* Discussion of the paper.

to make a second application after penetrating through the unæsthetized layer of dentine, in which case the current may be turned on more rapidly.

In the discussion of the paper, Dr. STEVENS suggested that in a very large cavity it might be advisable to place a platinum disk on the cotton, applying the electrode to the disk. A platinum point for the positive electrode is required for BLEACHING PURPOSES.

If a pulpless tooth, cleanse the cavity, fill the upper third of the canal, and apply, on cotton, to the exposed dentine, a 25-percent aqueous solution of pyrozone, made by shaking two parts of 25-percent ethereal pyrozone with one part of water, allowing the ether to evaporate. The tooth should be insulated by the rubber-dam, and each current used as the patient can bear, a stronger current and larger steps being possible in a pulpless tooth. An exposed pulp below softened dentine is shown by marked sensitiveness to low voltage.

DR. GILLETTE was asked in the discussion, if he would attempt to anæsthetize a tooth in which, while the pulp was not exposed, yet the tooth was aching violently. Dr. G. replied that he had done so repeatedly, though he thought it doubtful if it could be perfectly accomplished through a thick layer of dentine. He has anæsthetized exposed pulps when not very vascular or very large. Unless violently inflamed it will be so anæsthetized as to be painlessly removed. Usually, however, he merely benumbs the surface and then uses the syringe for injecting cocaine, as this can be done in five minutes while cataphoresis would require, perhaps, half an hour.

The report of the Committee on Practice, of the Dental Society State of New York,* while acknowledging the earlier use of the cataphoric process in producing anæsthesia of the soft tissues and its application to dentine, pays just tribute to Dr. H. W. Gillette, as the inventor of a rheostat which first enabled the electric current to be subdivided into such minute fractions the increase from the very smallest power to the highest is accomplished without any sensation of pain to the patient.

* Dental Cosmos, November, 1896.

The committee cautions against the use of too large an amount of cocaine as liable to be followed by subsequent hyperæsthesia, the duration of which is probably proportioned to the length of the stage of anæsthesia. They urge the co-operation of all employing cataphoric medication in investigating the possibilities of treating alveolar abscess, pyorrhœa alveolaris and dissolving uratic concretions on the roots of teeth by this method.

In the discussion of this report Dr Van Woert states, as the result of clinical experience, that cutting down the voltage, with a larger milliamperage, carries the medicament with it quite as effectively as a high pressure with small volume, the electric-current producing the after results (hyperæsthesia) and not the excess of cocaine as claimed in the committee report. If the current is cut down to the minimum a saturated solution of cocaine can be put into the tooth without any injurious effects following. A saturated solution, with from fifteen to twenty volts and three-fifths to one milliampere in volume, gives the best results in the least time.

In the treatment of pyorrhœa alveolaris, six minutes with ten volts, and two and one-half milliamperes so anæsthetizes the tissues that the pockets can be cut open without causing any pain, the sensibility of the roots also entirely disappearing. The surgical treatment is to be followed by the cataphoric application of one-half strength tincture of iodine, diluted with water.

A case of chronic alveolar abscess, a very stubborn sinus which had resisted other methods of treatment for months (four dentists having failed in curing it), yielded to one application of the current, and with an electrode, made by dipping a very fine wire into zinc, afterward coating it with chloride of zinc, forming a little ball at the end. The wire was insulated with paraffin and carried to the seat of the abscess. A current of ten volts was turned on and kept there for about five minutes. The mouth is as perfect to-day as can be. From these and like results Dr. Van Woert said he feels that cataphoresis in dentistry is going to be as necessary as the dental engine. To anæsthetize sensitive dentine in those difficult cavities along the buccal surfaces, well down under the gum margin, first anæsthetize the soft tissues in order to place the clamp painlessly. For this purpose Dr. Van Woert uses one of the little rubber polishing-cups, placing a pellet of cotton, saturated with cocaine, in the cup, passing the wire through the cup—five minutes' application of the current is sufficient. When the clamp is in position, anæsthetize the dentine by placing the cotton in the cavity holding it in position by a spring of German silver spring-wire, placed on the lugs of the clamp. To prevent breaking connection with the negative electrode, as is liable to be the case when it is held in the hand, especially by children, attach it to the wrist by some suitable device. By these means the patient can be left in the chair while the operator is attending to other duties, thus giving relief from the strain of standing by the chair holding the electrode in position.

Dr. L. E. Custer* discusses the causes of the sudden variation in the commercial currents when used for cataphoric purposes, resulting in severe shocks of pain to the patient. The fluctuation is usually a lowering of voltage, so quickly done that the ordinary volt-meter index has not time to record it. This fluctuation may be due to a variety of causes; the engines and dynamos may be too small for the work put upon them; the dentist may be getting his current from one of the ramifications of the wires supplying a large building, a neighbor turning on or shutting off the current, causing a sudden variation in the supply; or the wires may be crossed by the trolley, or be in contact with a water or gas-pipe, the practical conclusion being that the dentist should connect direct with the main line, should not use current for other purposes while producing cataphoresis, and should not be on the same circuit with large motors which operate intermittently. He should also see that his wiring is perfectly insulated and not in danger of a "ground." Unless these conditions can be secured, the battery-current is preferable to the 110-volt Edison current.

Dr. C. W. Jones, in a paper read before the Minnesota State Dental Association,† gives the results of his experience in

* Ohio Dental Journal, October, 1896.

the use of cataphoresis, from which he concludes that cocain-cataphoresis for sensitive dentine, though producing true and profound anæsthesia, in certain favorable cases, is quite limited in its application. For the extirpation of pulps, when nearly or lightly exposed, it is a complete success, and the apparatus necessary is worth having for these cases alone. He suggests the possibility of producing, by electrolysis, an enamel or vitreous coating for shallow cavities at the gingival margin of the gums; by the process of electro-gilding, by pressing particles of gold or other metallic substance, or some lime-salt deep into the dental tubes so as to form permanent parts of the tooth.

In the discussion of the paper Dr. LARRABEE said: It is a fact that cataphoresis is a living, vital force in the practice of dentistry, and has come to stay. I never have had failure in the use of cataphoresis since I put it in my office three months ago, * * * and I am very sure that any one who can manipulate the appliances properly will get from them the same results that I have.

In a paper on "Pulpitis," read by Dr. L. L. DAVIS before the Chicago Dental Society,* he said: Since the introduction of the electrical apparatus for cataphoresis I have attempted the removal of tooth-pulp by its use and met with good results. In the discussion of the paper Dr. P. J. Kester said: The discovery of cataphoresis has made it possible for the dentist to allay the pain of pulpitis as nothing else had. I can not agree with the essayist, however, that we can destroy a pulp successfully by cataphoresis.

At the same meeting Dr. C. P. Pruyn reported two cases of sloughing of gum-tissue following the use of the catephoric obtunder, fifteen volts, with ten percent cocain solution, the tissue being cooked, resembling a carbolic acid eschar, the tissue sloughing, leaving the root and alveolus exposed.

DR. A. I. F. BUXBAUM says†: From April 1st to July 15th I have used this method wherever the case demanded it, and at no time have I met with a failure to bring about the de-

† Dental Cosmos, November, 1896.

sired result. Not only has it proved a great source of gratification to my patients, but to me also has it brought relief to my nervous system. Cases have come into my office suffering intense pain from toothache and chronic pericementitis and alveolar abscess which I have relieved by direct application of cocain to the gum by means of cataphoresis, and in other cases when I desired rapid absorption, I have applied tincture of iodin the same way, and obtained excellent results. The danger of cataphoresis lies, not in its application to the patient, but in the ease and boldness with which the dentist can operate.

If he be careless he could easily penetrate or expose the pulp, not eliciting any pain; he might be careless and not protect the pulp. A great advantage found in cataphoresis is all freedom from pain when finishing off a filling with disks, strips, stones, etc. I have found the larger the cavity and the softer the dentine the more readily will cocain be transmitted.

NEW APPLIANCES IN BLEACHING TEETH BY CATAPHORESIS.

In a clinic, by Dr. Hollingsworth, of Santa Barbara, California, read before the New Jersey State Dental Society,* the tooth to be bleached was completely surrounded by an especially devised rubber nipple, secured by silk ligatures. Opposite the mouth of the nipple was inserted a small glass tube containing a platinum coil. The terminal end of the tube was provided with a metallic tip with tapered opening through which the bleaching agent (pyrozone, twenty-five percent), was introduced, and into which fitted a metal plug forming the terminal of the wire leading to the electric supply. A special device was used to facilitate the introduction of the pyrozone. This consisted of two connected rubber bulbs having a common nozzle duplex in character, that is, the longer nozzle of one bulb passed through the other bulb and its nozzle, preserving separately the function of each bulb. The outer bulb was filled with the liquid in the usual manner. The second bulb was then slightly compressed and the common nozzle inserted into the tube-opening which it closely fitted. The compressed bulb was then released, causing a collapse of the nipple. The bulb filled with pyrozone was then compressed sufficiently to fill the nipple and tube, any excess being drawn into the lower dry bulb. The tooth being thus surrounded by the bleaching agent, the syringe or injector was removed and the plug forming the terminal of the positive wire inserted, the cathode being placed in the hand of the patient. Experience leads the operator to believe that the bleaching action of the agent continues for several hours after cataphoretic action has ceased. Dr. Hollingsworth points out the importance of filling root-canals before applying the current, which would otherwise escape through the apical foramen.

Dr. W. B. Ames, in the *Western Journal*,* suggests the danger of *leakage of current* through metal fillings if not insulated. He says: You might open one surface of a tooth through which you are trying to obtund the dentine and have a filling already in the same tooth which would reach so deeply into the dentine that when your current enters the newly-opened cavity it will go through that filling and through other tubuli of the tooth, and you will get amost no result.

Dr. Eaton† spoke of *the pain* caused by cataphoresis in teeth having fillings in them, to which Mr. Wheeler replied that the one thing to do in such cases was to insulate the fillings. Mr. Wheeler uses sandarac or shellac varnish as an insulator. Dr. Gillette uses "Gilbert's Temporary Stopping."

Dr. Ames,‡ in the *Western Dental Journal*, suggests that with an electrode of zinc or some other metal which is affected by the electric current and the salts of the metal thrown out by electrolysis, there is a possibility of those salts being conducted into the tissues and being used in very salutary ways in a large number of cases, particularly in the treatment of pyorrhœa alveolaris. He says: I can introduce an electrode of zinc into a pyorrhœa pocket and get astonishing results from the salts of zinc, and I say the application is almost unlimited.

At the meeting of the American Dental Association, August, 1896,* Dr. Cassidy, Chairman Section 5, read a report on the

* Ohio Dental Journal, November, 1896.

† Ohio Dental Journal, October, 1896.

‡ Ohio Dental Journal, November, 1896.

"Process of Cataphoresis," the laws of electrolysis, and the various combinations of drugs as cataphorics.

Of "Eucanie," the newest local anæsthetic, he said: As a cataphoric obtundent it seems to act favorably, although more experiments will have to be made before its virtues, in this direction, are accepted as satisfactory. It has a decided advantage over cocain in that it is probably uniform in its action, other conditions being equal, on account of its immediate derivation from pure chemicals instead of indirectly from vegetable sources.

DR. L. P. BETHEL read a paper on "Lining Root-Canals with Nitrate of Silver," introduced by the aid of cataphoresis. (See DENTAL REGISTER, September, 1896, page 417.)

* International Dental Journal, November, 1896.

